# Prevalence of Impulse Control Disorders in Bipolar Patients

**DOI:** 10.1192/j.eurpsy.2026.11079

**Published:** 2026-07-31

**Authors:** F. Sahnoun, S. Ellouze, R. Chakchouk, S. Kotti, N. Halouani, M. Turki, J. Aloulou

**Affiliations:** Psychiatry “B” Department, Hédi Chaker University Hospital, Sfax, Tunisia

## Abstract

**Introduction:**

Impulse Control Disorders (ICDs) represent a frequent and often overlooked comorbidity in bipolar disorder. They may significantly affect the course of the illness, and thus treatment efficacy. Comparing their prevalence between bipolar patients and healthy individuals provides valuable insights into the specific vulnerability of this population.

**Objectives:**

The aim of our study was to assess impulsivity and impulse control disorders (ICDs) in patients with bipolar disorder compared with a matched control group.

**Methods:**

We conducted a case–control study at the Psychiatry “B” Department of Hédi Chaker University Hospital. Patients with bipolar disorder diagnosed according to DSM-5 criteria and evaluated during the euthymic phase were included. Age- and sex-matched healthy controls were also recruited. ICDs were assessed using the modified Minnesota Impulsive Disorders Interview (mMIDI). Intermittent Explosive Disorder was evaluated separately with the Intermittent Explosive Disorder Screening Questionnaire (IED-SQ). Impulsivity was measured using the Barratt Impulsiveness Scale, version BIS-11

**Results:**

A total of 72 patients with bipolar disorder and 72 healthy controls were included. The mean age was 44.13 years for cases and 42.82 for controls. The sex ratio (F/M) was 1.88 in both groups.

The mean BIS-11 score was 57.07±11.18 in patients versus 45.36±9.68 in controls. A marked level of impulsivity was observed in 12.5% of cases and in 2.8% of controls.

Among cases, 33.3% had at least one impulse control disorder (ICD) compared to 18.1% of controls. The most frequent ICDs in patients were intermittent explosive disorder (12.5%) and hypersexuality (12.5%), followed by pathological gambling (6.9%), kleptomania, and pyromania (4.2%). In controls, intermittent explosive disorder (13.9%) and pathological gambling (5.6%) were most common, followed by trichotillomania (2.8%). Overall, ICDs were significantly more prevalent among bipolar patients than among controls (p = 0.001).

**Conclusions:**

ICDs are considerably more prevalent among individuals with bipolar disorder than in the general population. Systematic screening for these disorders should be integrated into the clinical assessment of bipolar patients to enable early identification and optimize management and outcomes.

**Disclosure of Interest:**

None Declared

